# Integrated Machine Learning and Structure-Based Virtual Screening Identifies Natural Product Targeting 50S Ribosome Inhibitory Activity Against *Cutibacterium acnes*

**DOI:** 10.3390/molecules30224433

**Published:** 2025-11-16

**Authors:** Jixing Liu, Henry H. Y. Tong, Hang Zheng, Miriam Iun Fan Lei, Shu Li

**Affiliations:** 1 Centre for Artificial Intelligence Driven Drug Discovery, Faculty of Applied Sciences, Macao Polytechnic University, Macao 999078, China; p2316804@mpu.edu.mo (J.L.); henrytong@mpu.edu.mo (H.H.Y.T.); 2 Faculty of Health Sciences and Sports, Macao Polytechnic University, Macao 999078, China; 3 School of Chemical Biology and Biotechnology, Peking University Shenzhen Graduate School, Shenzhen 518055, China; hzheng@pku.edu.cn

**Keywords:** acne vulgaris, *Cutibacterium acnes*, 50S ribosomal subunit, machine learning, docking, natural products, tripterin

## Abstract

Acne vulgaris is a prevalent inflammatory disease of the pilosebaceous unit in which *Cutibacterium acnes* (*C. acnes*) contributes to lesion initiation and persistence, supporting antibacterial interventions as a component of clinical management. Given the essential role of the 50S large ribosomal subunit—particularly 23S rRNA sites in the peptidyl transferase center and nascent peptide exit tunnel—in *C. acnes* protein synthesis and viability, targeting the 50S offers an effective path to lead discovery for acne treatment. Here, we present an integrated computational–experimental workflow to identify anti-*C. acnes* candidates from a 186,659-compound natural product library. Curated 50S/23S ligands trained and validated two ML-QSAR regression models built on different molecular fingerprints (MACCS keys and PubChem 2D) to predict anti-*C. acnes* activity and rapidly triage the library. Compounds were further screened by ADMET filtering and structure-based docking to 23S rRNA pockets, followed by cluster and interaction analysis. Among six experimental hits, three compounds exhibited MICs against *C. acnes* of ≤8 μg/mL, with tripterin, a pentacyclic triterpenoid, being the most potent (0.5–2 μg/mL across two acne-relevant strains). Collectively, these results indicate that a 50S ribosomal-focused, multistage computational screening workflow, integrated with in vitro assays, efficiently prioritizes compounds with quantifiable anti-*C. acnes* activity across a broad range of natural products.

## 1. Introduction

Acne vulgaris is a common chronic inflammatory disorder of the pilosebaceous unit [1]. Its pathogenesis involves the interplay of increased sebum production, follicular hyperkeratinization, shifts in the skin microbiome, and innate and adaptive immune responses [2]. *Cutibacterium acnes* (*C. acnes*) is highly abundant in lipid-rich follicles and is closely implicated in lesion initiation and persistence. *C. acnes* lipases hydrolyze triglycerides to free fatty acids that perturb barrier function and keratinization [3]; cell wall components and metabolites activate pattern-recognition pathways (e.g., TLR2, NLRP3) to drive IL-1β and IL-8 production; and biofilm formation and strain-level heterogeneity have been associated with clinical phenotypes and treatment responses [4,5]. Clinical experience and guidelines indicate that acne vulgaris can be mitigated by interventions that suppress or eliminate *C. acnes*, thereby reducing inflammation and new lesions. For example, Benzoyl peroxide releases reactive oxygen species within the follicular environment and rapidly lowers *C. acnes* counts [6,7]. Topical clindamycin and erythromycin, and oral tetracyclines such as doxycycline and minocycline, inhibit bacterial protein synthesis by targeting the ribosome [8,9] and are widely used across disease severities. These examples support a rationale for discovery strategies that directly inhibit *C. acnes* as a component of acne vulgaris management.

**Figure 1:**
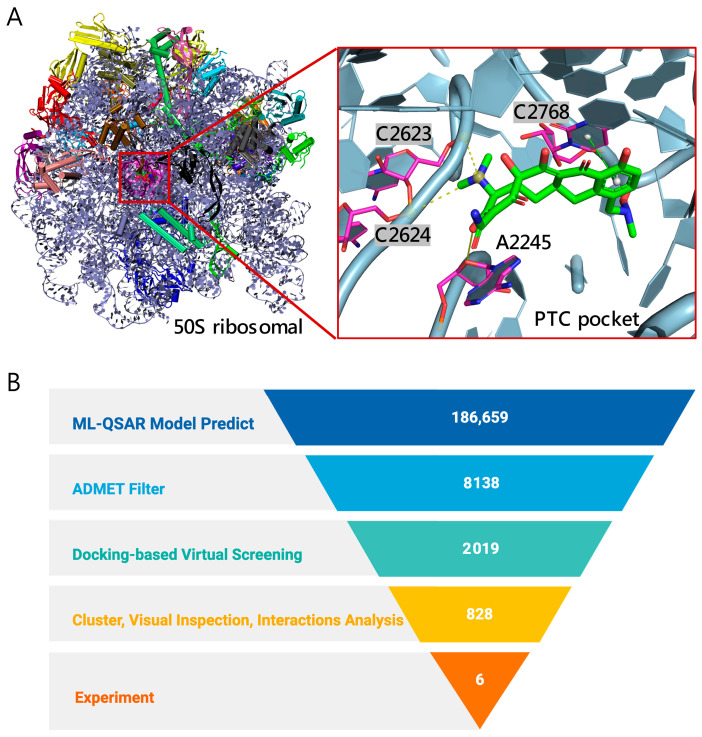
Overview of the screening workflow and binding site. (**A**) Structure of the *C. acnes* 50S ribosomal subunit (PDB ID: 8CVM) [15], highlighting the PTC pocket (magenta). The inset shows the docked pose of a representative antibiotic sarecycline (green) within the PTC, with nearby RNA nucleotides labeled (A2245, C2623, C2624, C2768). (**B**) 50S ribosomal subunit-focused screening funnel for anti–*C. acnes* discovery. From 186,659 natural products, ML-QSAR enrichment, ADMET filtering, docking, and clustering/interaction analysis prioritized candidates, leading to 6 compounds selected for experimental validation.

The bacterial 50S ribosomal subunit—composed of 23S rRNA, 5S rRNA, and multiple associated proteins—contains essential functional regions for translation, notably the peptidyl transferase center (PTC) and the nascent peptide exit tunnel (NPET) [10]. These regions are formed primarily by 23S rRNA and constitute principal binding sites for several antibiotic classes (e.g., macrolides, lincosamides, amphenicols, streptogramins, and oxazolidinones) that inhibit protein synthesis (Figure 1A) [9,10,11,12]. Local structural differences in the 50S subunit across bacterial species can influence ligand binding modes and affinities, providing a structural basis for identifying compounds with activity against *C. acnes* [13,14]. Accordingly, focusing on 23S rRNA sites within the *C. acnes* 50S subunit is a biologically and pharmacologically well-founded approach for antibacterial hit discovery.

However, conventional experimental discovery routes have been inefficient for fully exploiting this target. High-throughput screening is resource-intensive and often rediscovers known scaffolds, while derivatizing existing antibiotics seldom yields genuinely new chemotypes [16]. This creates a methodological gap: the need to efficiently explore vast, diverse chemical space. An integrated strategy that couples ligand-based and structure-based computational methods can improve enrichment and hit quality in large libraries. Machine learning quantitative structure–activity relationship (ML-QSAR) models leverage known actives and inactives to estimate activity propensity and enable rapid triage of unrelated chemotypes, thereby reducing downstream computational and experimental burden. Molecular docking provides atomistic assessments of geometric complementarity and key interaction networks within defined binding pockets [17], supporting fine-grained prioritization among high-scoring candidates [18]. In parallel, early absorption, distribution, metabolism, excretion, and toxicity (ADMET) profiling and chemical diversity control help enhance developability and minimize redundancy.

In this study, we developed a 50S/23S-anchored, integrated computational–experimental workflow to identify anti–*C. acnes* leads from a large set of natural products. Curated 50S/23S ligands informed two fingerprint-based ML-QSAR models, which were validated by stratified cross-validation and external testing and then used for rapid, large-scale prioritization. Predicted actives underwent ADMET filtering, followed by structure-based docking to 23S rRNA PTC pocket in a *C. acnes* 50S model and interaction analysis; fingerprint clustering preserved chemical diversity for down-selection. This pipeline advanced a small, diverse set to testing, yielding six hits, three with minimum inhibitory concentrations (MICs) ≤ 8 μg/mL, including tripterin (0.5–2 μg/mL across two acne-relevant strains).

## 2. Results and Discussion

The screening process targeting 23S rRNA sites within the 50S subunit to identify potent anti–*C. acnes* compounds comprised five stages (Figure 1B). First, we assembled a library of 186,659 natural products from the Zinc Database [19]. Second, two ML-QSAR models—one using MACCS fingerprints and the other using PubChem 2D fingerprints—were applied in consensus mode; only compounds passing both models proceeded to ADMET filtering, yielding 8138 candidates. Third, ADMET properties were evaluated with QikProp, resulting in 2019 drug-like molecules. Fourth, structure-based docking against *C. acnes* 50S PTC pocket shortlisted 828 compounds with predicted binding energies better than −9 kcal/mol. Fifth, fingerprint-based clustering reduced these to 60 chemotype groups; after interaction analysis and compound availability checks, six representatives were selected for experimental validation.

### 2.1. Rapid Screening of 50S Ribosomal Subunit Inhibitors Using Two ML-QSAR Models

We curated 353 compounds with reported 50S ribosomal inhibition from ChEMBL [20]. Two complementary ML-QSAR models were built to predict anti–*C. acnes* activity using distinct molecular fingerprint representations: one based on MACCS keys and the other on PubChem 2D fingerprints. Feature selection was performed by removing low-variance features (variance < 0.1) and highly correlated pairs (Pearson correlation > 0.8), yielding 46 features for the MACCS set and 41 for the PubChem set. Data were split 8:2, resulting in 282 molecules for training and 71 for testing in each dataset.

To determine the best-performing approach, we evaluated three representative algorithms—artificial neural network (ANN), random forest (RF), and support vector machine (SVM)—on both fingerprint sets. For each model, we computed a comprehensive set of performance metrics, including root mean squared error (RMSE), mean squared error (MSE), mean absolute error (MAE), Pearson correlation coefficient (Rp), and 10-fold cross-validation statistics. Moreover, we further processed the fingerprint representations using principal component analysis (PCA) to project the descriptors onto the first two principal components to identify outliers. Molecules lying outside the main clusters were removed, after which the models were retrained using grid search and cross-validation. This PCA-based outlier removal was applied solely to the training set for quality control and is distinct from the diversity-based clustering used in screening.

**Figure 2:**
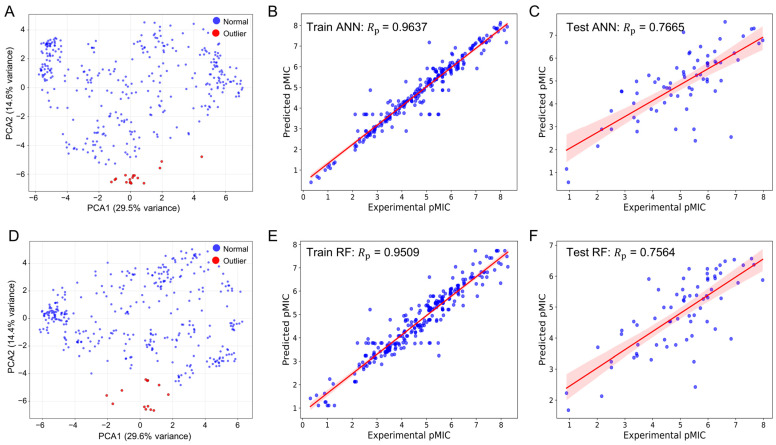
Predicted versus experimental pMIC following PCA-guided outlier curation. (**A**) PCA of MACCS fingerprints highlighting outliers used for curation. (**B**) MACCS-based ANN, training set (Rp = 0.9637). (**C**) MACCS-based ANN, test set (Rp = 7665). (**D**) PCA of PubChem descriptors highlighting outliers used for curation. (**E**) PubChem–based RF, training set (Rp = 0.9509). (**F**) PubChem–based RF, test set (Rp = 0.7564). Blue markers denote individual compounds; red lines indicate least-squares regressions with 95% confidence intervals. All models were optimized via grid search under a consistent 10-fold cross-validation scheme.

As shown in Figure 2A–C and Table S1, the optimized MACCS fingerprint-driven ANN model demonstrated excellent predictive capability after removing 18 outliers, achieving strong performance metrics. For the training set, it yielded a Rp = 0.9637, RMSE = 0.4395, MSE = 0.1932, and MAE = 0.2647. For the test set, it maintained good generalizability with Rp = 0.7665, RMSE = 1.0409, MSE = 1.0836, and MAE = 0.7980, supported by a 10-fold cross-validation RMSE = 1.0601.

Similarly, the optimized PubChem fingerprint-driven RF model exhibited robust performance after removing 13 outliers (Figure 2C–E and Table S2). The model achieved comparable training performance with Rp = 0.9513, RMSE = 0.5329, MSE = 0.2840, and MAE = 0.3882, while maintaining consistent test performance (Rp = 0.7570, RMSE = 1.0458, MSE = 1.0936, MAE = 0.8107) and a superior 10-fold cross-validation RMSE of 0.9666. These complementary models provide a foundation for comprehensive virtual screening by capturing different aspects of structure–activity relationships.

Then, we applied both finalized models to 186,659 natural products and implemented a stringent consensus rule: only compounds predicted to meet pMIC ≥ 6 (where $pMIC={-log}_{10}\left(MIC\right)$, corresponding to an MIC ≤ 1 × 10^−6^ µg/mL) by both models were retained. This dual-threshold consensus yielded 8138 concordant hits with consistently high predicted activity, reducing model-specific biases and curbing false positives.

### 2.2. Feature Importance Analysis Reveals Key Molecular Determinants of Antimicrobial Activity

Model interpretability was interrogated using complementary approaches—variance importance in projection (VIP) [21], correlation matrix analysis and Shapley Additive exPlanations (SHAP) [22]—to quantify global importance and instance-level attributions.

**Figure 3:**
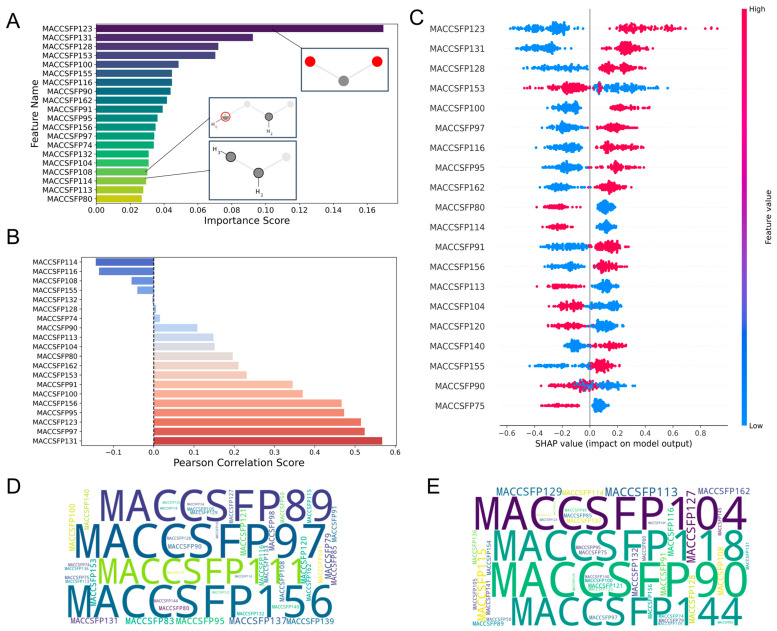
Visualization of feature importance analysis for the MACCS fingerprint-driven ANN-QSAR model. (**A**) Top 20 features from VIP analysis. (**B**) Pearson correlation analysis. (**C**) SHAP importance analysis. Tanimoto similarity-based clustering analysis of (**D**) highly active and (**E**) low active molecules from the MACCS fingerprint dataset.

For the MACCS fingerprint-driven ANN model, comprehensive feature importance analysis revealed several key determinants of biological activity. As shown in Figure 3A, MACCSFP123 (oxygen-carbon-oxygen linkage, O-C-O) and MACCSFP131 (multiple heteroatoms with hydrogen, QH > 1) emerged as high-contributing features promoting antimicrobial activity, whereas MACCSFP114 (ethyl substituent, CH3CH2A) exhibited a lower variance threshold score, indicating reduced importance. Correlation analysis (Figure 3B) confirmed positive correlations between MACCSFP123, MACCSFP131, and pMIC values while revealing negative correlations for MACCSFP114 and MACCSFP104 (heteroatom-CH2 linkage, QHACH2A). The SHAP analysis, presented in Figure 3C, corroborated these findings by showing high positive SHAP values for MACCSFP131 and MACCSFP97 (extended nitrogen-oxygen chain, NAAAO) and negative values for MACCSFP114 and MACCSFP104. Word cloud visualizations (Figure 3D,E) further confirmed a predominance of MACCSFP97 in high-activity molecules and enrichment of MACCSFP104 in low-activity compounds. Detailed chemical definitions for all fingerprint features are provided in Table S3.

**Figure 4:**
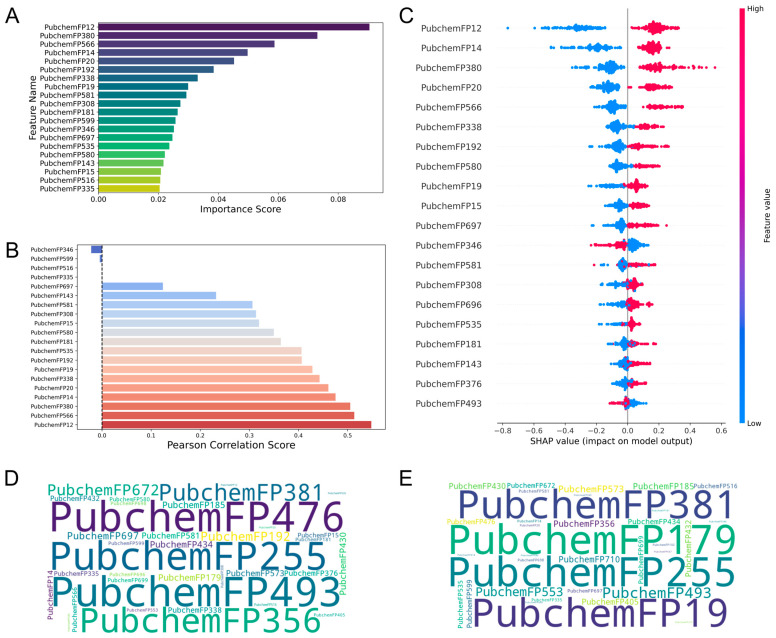
Visualization of feature importance analysis for the Pubchem fingerprint-driven RF-QSAR model: (**A**) Top 20 features from VIP analysis. (**B**) Pearson correlation analysis. (**C**) SHAP importance analysis. Tanimoto similarity-based clustering analysis of (**D**) highly active and (**E**) low active molecules from the Pubchem 2D fingerprint dataset.

The PubChem fingerprint-driven RF model revealed complementary molecular determinants that enhance the comprehensiveness of feature analysis. PubchemFP12 (≥16 carbons) and PubchemFP380 (carbon with two oxygen neighbors, C(~O)(~O)) demonstrated high variance threshold scores as beneficial features, while PubchemFP346 (carbon with C, H, and O neighbors, C(~C)(~H)(~O)) showed adverse effects on activity (Figure 4A). Correlation and SHAP analyses (Figure 4B,C) consistently identified PubchemFP12 and PubchemFP380 as positively correlated with biological activity. Word cloud analysis (Figure 4D,E) revealed that high-activity molecules were dominated by PubchemFP493 (aromatic ketone, O=C-C:C) and PubchemFP476 (enol ether pattern, C-O-C=C), while low-activity compounds were enriched with PubchemFP179 (saturated 6-membered carbon ring) and PubchemFP255 (simple aromatic ring). Comprehensive chemical definitions for all fingerprint features are provided in Table S3.

In total, these analyses of molecular fingerprints suggest that higher structural complexity is positively associated with activity against the 50S ribosomal subunit: larger carbon frameworks (≥16 carbons) and multiple ring systems favor selectivity, consistent with tripterin’s pentacyclic scaffold. Oxygen-rich functionalities also appear beneficial—ether/ester linkages and carbons adjacent to two oxygens likely support hydrogen-bonding networks with the rRNA backbone. Conjugated motifs are preferred, with aromatic ketones showing positive correlation and simple aromatics showing negative correlation, indicating that conjugated carbonyl systems enhance binding affinity. In contrast, certain features correlate with reduced activity, including simple alkyl substituents (e.g., ethyl groups) and simple alcohol patterns, suggesting that nonpolar or weakly interactive fragments may impair key site interactions.

### 2.3. ADMET Filtering and Docking Screen of Natural Compounds

Starting from 8138 candidates prioritized by the consensus ML-QSAR models, we performed an ADMET-focused triage using QikProp to retain compounds with oral drug-like and safety-compatible profiles. The retained set collectively met the following ranges: molecular weight 130–725 Da; polar surface area (PSA) 7–200 Å^2^; hydrogen-bond donors 0–6 and acceptors 2–20; predicted human oral absorption > 25% for all compounds; octanol–water partition coefficient (logP) from −2 to 6.5; 1–8 putative metabolic soft spots; predicted hERG liability (QPlogHERG) > −5; and CNS permeability scores between −2 and +2. These criteria ensured a balanced solubility–permeability profile, manageable metabolic liabilities, and reduced cardiotoxic/CNS risks, yielding 2019 ADMET-qualified candidates for structure-based screening.

For target-focused docking, the *C. acnes* 50S ribosomal subunit (PDB 8CVM) [15] was used. Protein preparation comprised assignment of protonation and tautomeric states at physiological pH, hydrogen-bond network optimization, and restrained minimization. Protocol performance was evaluated by redocking the cognate ligand sarecycline with Schrödinger Glide v2018. The experiment reproduced the cryo-EM binding mode with an RMSD of 0.66 Å (Figure S1), demonstrating reliable pose recovery and supporting the suitability of this docking setup for prospective screening on the 50S ribosomal subunit. The redocking score (−9.0 kcal/mol) was taken as the decision threshold. Prospective docking of the 2019 ADMET-qualified molecules then shortlisted 828 compounds scoring at or below this threshold, suggestive of high-affinity engagement within the 50S binding pocket.

To preserve chemical breadth and minimize redundancy, the 828 top-scoring ligands were clustered by Tanimoto similarity (cutoff = 0.8) using 2D fingerprints, yielding 60 non-redundant clusters. Six structurally diverse representatives were selected based on binding affinity, interaction profiles, and commercial availability (Table 1). This diversity-first approach maximizes initial chemical space coverage; clusters showing superior activity in bioassays are prioritized for subsequent member testing.

### 2.4. Experimental Validation Confirms Tripterin as the Most Potent Anti-C. acnes Candidate

Antimicrobial susceptibility testing validated the computational predictions and identified tripterin as the lead compound with exceptional anti-*C. acnes* activity. All six computationally selected compounds were systematically evaluated against *C. acnes* strains ATCC 6919 and ATCC 11827 using standardized broth microdilution assays. As detailed in Table 2, tripterin exhibited the most promising antimicrobial activity, demonstrating potent inhibition with MIC values of 2 µg/mL and 0.5 µg/mL against the respective strains, representing only a 4- to 8-fold reduction in potency compared to the clindamycin reference standard.

Two additional compounds showed moderate antimicrobial activity, with kaurenoic acid and EC23 both achieving MIC values of 8 µg/mL against both tested strains, indicating consistent but less potent activity. In contrast, three compounds demonstrated limited antimicrobial potential with MIC values exceeding 64 µg/mL: glucotropaeolin potassium, carbenoxolone disodium, and 3-oxo-5β-cholanoic acid. The clindamycin positive control performed as expected, exhibiting MIC values of 0.25 µg/mL and 0.125 µg/mL against ATCC 6919 and ATCC 11827, respectively, confirming the validity of the experimental protocol and providing a benchmark for activity comparison.

### 2.5. Computational Analysis Suggests Tripterin’s PTC-Centered Interactions on 23S rRNA

**Figure 5:**
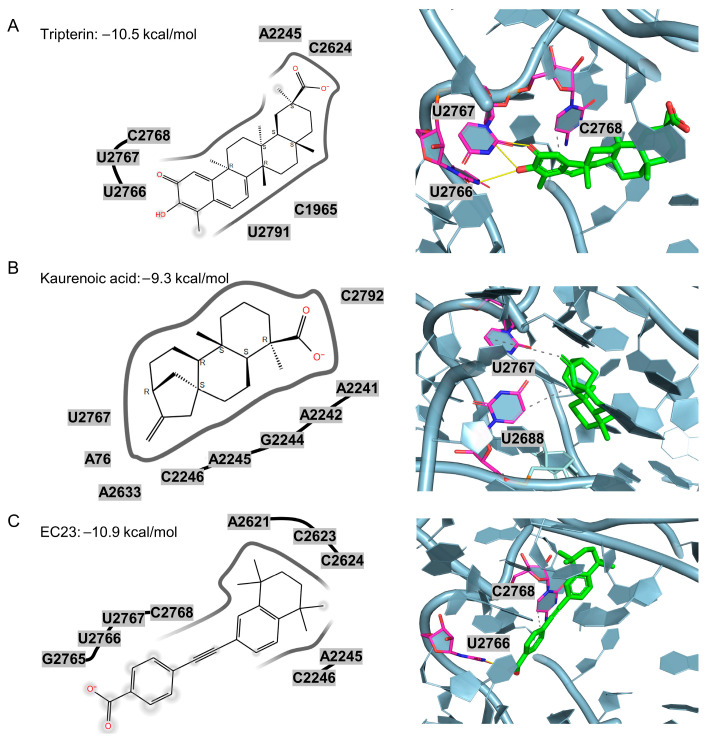
2D and 3D docking interactions of three anti–*C. acnes* compounds within the PTC pocket of the 50S ribosomal subunit. (**A**) Tripterin (Glide score −10.5 kcal/mol) engages nucleotides near U2766, U2767 and C2768 via hydrogen-bond and π–π interactions. (**B**) Kaurenoic acid (−9.3 kcal/mol) forms contacts around U2767 and U2688. (**C**) EC23 (−10.9 kcal/mol) aligns along the pocket and interacts with U2766 and C2768. The protein is shown as cartoon/surface, nearby RNA bases are labeled, and ligands are depicted in green with key neighboring residues highlighted in magenta.

Docking and structural analysis predict that tripterin engages the *C. acnes* 50S ribosomal subunit PTC with favorable binding geometry, consistent with its antimicrobial activity. As shown in Figure 5A, the top-ranked pose forms three key hydrogen bonds with donor–acceptor distances of 3.53 Å to U2766, and 3.44 Å and 3.12 Å to U2767. Oxygen atoms from tripterin’s hydroxyl groups serve as both donors and acceptors, collectively stabilizing the ligand–target complex.

Beyond hydrogen bonding, tripterin demonstrated extensive hydrophobic interactions that contribute to binding stability and specificity. The compound established hydrophobic contacts with fourteen rRNA residues: U2766, U2767, G2687, U875, C2622, C2623, C2624, C2625, A2242, A2245, A837, G2763, G2790, U2791, and C2792, creating a stable binding environment within the ribosomal active site. Importantly, computational analysis confirmed that tripterin’s molecular profile aligns favorably with the previously identified beneficial fingerprint features (MACCSFP123, PubchemFP12, PubchemFP380) while successfully avoiding detrimental structural motifs (MACCSFP114, MACCSFP104, PubchemFP346). This molecular feature alignment provides strong support for the validity of our ML-QSAR predictions and explains tripterin’s superior experimental performance among the tested candidates.

Docking also predicts that EC23 and kaurenoic acid engage the PTC pocket through hydrogen-bond and hydrophobic interactions. EC23 forms a strong hydrogen bond to U2766 with a donor–acceptor distance of 2.98 Å, while kaurenoic acid primarily contacts G2687, U2688, and U2767. Comprehensive interaction data for all six tested compounds, including hydrogen bond distances, donor angles, and hydrophobic contacts, are presented in Table S4. As shown in Figure 5, although tripterin, kaurenoic acid, and EC23 each contain a carboxyl group, their orientations within the PTC pocket differ. In EC23, the carboxylate projects toward the G2765–C2768 region, whereas in tripterin and kaurenoic acid, it aligns closer to A2245 and A2241. Notably, the carboxyl group in tripterin and kaurenoic acid is appended to a polycyclic, steroid-like scaffold, while in EC23 it is attached to an aromatic ring. This suggests that engagement of the G2765–C2768 region may favor sterically smaller, aromatic substituents, whereas the polycyclic scaffolds impose greater three-dimensional bulk. In addition, tripterin’s interactions with U2766 and U2767 are supported by the planar salicylaldehyde-like ring, which may facilitate stacking and hydrogen-bonding geometry. Thus, despite the shared carboxyl functionality, the local chemical context and spatial presentation of the group differ across these chemotypes, leading to distinct binding modes within the PTC.

Binding modes of the three inactive compounds (carbenoxolone disodium, 3-oxo-5β-cholanoic acid, and glucotropaeolin potassium) are shown in Figure S2. Although all three fit reasonably within the 50S PTC pocket, they lack well-oriented aromatic substituents to engage the U2766–U2767 region, in contrast to tripterin and EC23. This suggests that directional, appropriately positioned aromatic motifs may be required to optimize PTC interactions and enhance activity.

## 3. Discussion

By integrating machine learning and structure-based virtual screening, tripterin was successfully identified as a promising anti-*C. acne* therapeutic. Tripterin, a pentacyclic triterpenoid from *Tripterygium wilfordii*, targets *C. acnes* through putative inhibition of the 50S ribosome.

The developed ML-QSAR models demonstrated robust predictive capabilities. The MACCS fingerprint-driven ANN model and the PubChem fingerprint-driven RF model achieved high training correlations (0.9637 and 0.9513, respectively) and good test correlations (0.7665 and 0.7570, respectively). These results indicate that the different molecular representations captured distinct structure–activity relationships, thereby providing comprehensive coverage of the relevant chemical space. Quality control measures, including outlier detection and dual-threshold screening, enhanced model reliability and achieved a 23-fold enrichment of candidates, demonstrating the efficiency of the computational approach.

Interpretability analysis successfully identified key molecular determinants governing anti-*C. acnes* activity. The identified beneficial features—such as MACCSFP123 (oxygen-carbon-oxygen linkage) and PubchemFP12 (large molecular size)—and detrimental features—such as MACCSFP114 (simple ethyl substituent) and PubchemFP346 (simple alcohol pattern)—offer valuable guidance for rational inhibitor design (Table S3). Tripterin’s favorable molecular profile, which aligns with beneficial features while avoiding detrimental motifs, likely accounts for its superior experimental performance and supports the validity of our computational predictions.

Tripterin exhibited notable antimicrobial activity against *C. acnes* (MIC: 0.5–2 μg/mL), demonstrating a 4- to 8-fold reduced potency compared to clindamycin. However, its clinical significance may be substantially enhanced considering the increasing clindamycin resistance (>22% in many regions) [23] and tripterin’s unique dual-action mechanism. The compound’s established anti-inflammatory properties arise from its targeted inhibition of a key signaling pathway. Specifically, it inhibits the nuclear factor kappa-light-chain-enhancer of activated B cells (NF-κB) pathway [24].

In acne vulgaris, *C. acnes* activates NF-κB signaling in sebaceous and immune cells. This activation upregulates pro-inflammatory cytokines, such as Tumor Necrosis Factor-alpha (TNF-α) and Interleukin-6 (IL-6), which in turn drive inflammation [25]. Tripterin’s ability to suppress NF-κB activation theoretically enables it to simultaneously target both bacterial colonization and the inflammatory cascade [26]. This comprehensive approach addresses acne’s multifactorial pathogenesis more effectively than single-target agents and could eliminate the need for combination regimens and their associated adverse effects [27].

Tripterin’s structural features likely facilitate specific interactions with the ribosomal target, contributing to its antimicrobial efficacy. Furthermore, its origin from *Tripterygium wilfordii*—a plant with a history of use in traditional Chinese medicine for its anti-inflammatory properties—lends additional support to its therapeutic potential [28]. This dual mechanism represents a substantial improvement over current therapies, which often fail to address the complex interplay between bacterial colonization, sebaceous gland dysfunction, and inflammation [29].

The molecular docking analysis predicts specific interactions between tripterin and the *C. acnes* 50S ribosomal subunit, including three critical hydrogen bonds within the peptidyl transferase centre: one with nucleotide U2766a and two with nucleotide U2767a, involving oxygen atoms from tripterin’s hydroxyl groups acting as both hydrogen bond donors and acceptors [30]. This predicted binding pattern presents a mechanistic hypothesis that could theoretically circumvent existing resistance mutations affecting macrolide and lincosamide binding sites (A2058 and A2059) [31]. The compound’s rigid pentacyclic framework and quinone methide functional group appear geometrically complementary to the binding site. This complementarity could create a stable drug-target complex that interferes with protein synthesis.

Collectively, these findings validate the utility of an integrated computational approach for accelerating natural product-based antimicrobial discovery [32]. Similar ML-QSAR and docking workflows have successfully identified potent natural product inhibitors in other therapeutic areas, including tyrosinase inhibition for dermatological applications [33]. The methodology is readily adaptable to other therapeutic targets and is particularly valuable for addressing antimicrobial resistance [34]. Tripterin’s natural origin offers several development advantages, including established use profiles and potential for sustainable production [35]. Its predicted pharmacokinetic properties suggest compatibility with topical formulations, though comprehensive ADMET evaluation remains necessary [36].

A key limitation of this study is the absence of direct evidence that the active compounds engage the 50S ribosomal subunit to elicit antibacterial effects. Although docking suggests plausible 50S binding, MIC-based phenotypic assays alone cannot discriminate on-target activity from alternative mechanisms, such as membrane perturbation or inhibition of non-ribosomal pathways. Consequently, the proposed 50S mechanism should be regarded as a working hypothesis rather than a demonstrated mode of action. Future studies could address this gap with target-proximal assays, including cell-free protein synthesis inhibition to assess ribosome-directed activity and ribosome binding experiments to measure target engagement. Resistance profiling (e.g., selection and sequencing of resistant mutants to identify mutations in 23S rRNA or 50S ribosomal proteins) could further substantiate the mechanism, and structural characterization (e.g., cryo-EM or chemical footprinting) could offer orthogonal confirmation. Beyond mechanism, in vivo efficacy and toxicology studies are the essential next steps to evaluate translational potential once target engagement is established.

It is also noted that docking hits were subjected to similarity-based clustering, and one representative per cluster was selected for initial biological evaluation, with selection informed by docking score, interaction profiles, and commercial availability. This representative sampling can, in principle, miss promising candidates within or across clusters—particularly compounds with unreported or previously unrecognized 50S ribosomal inhibition. Nonetheless, the diversity-preserving design was chosen to balance screening efficiency with broad chemical-space coverage under constrained experimental resources, thereby maximizing the probability of identifying chemotypes with tractable activity. The outcome supports this rationale: three actives originated from distinct clusters—tripterin (cluster 25, MIC 0.5–2 μg/mL), kaurenoic acid (cluster 33, MIC 8 μg/mL), and EC23 (cluster 19, MIC 8 μg/mL). Guided by these results, further work could prioritize the prospective expansion within active clusters (e.g., additional members of cluster 25).

## 4. Materials and Methods

### 4.1. Data Curation

The ML-QSAR model was developed to identify inhibitors of the 50S ribosomal subunit [37]. To build this model, a systematic data curation strategy was implemented, focusing on compounds with demonstrated ribosomal targeting mechanisms. The ChEMBL database [38] was queried using specific criteria: (1) compounds known to inhibit the bacterial 50S ribosomal subunit, and (2) established ribosome-targeting antibiotics effective against Gram-positive bacteria, including macrolides, lincosamides, and chloramphenicol derivatives. This targeted approach yielded 353 compounds with their chemical structures as Simplified Molecular Input Line Entry System (SMILES) strings and bioactivity data as MIC values. To ensure dataset homogeneity, compounds with non-ribosomal mechanisms of action, such as retinoids, anti-inflammatory agents, and topical antiseptics, were excluded. For normalization within the ML-QSAR model and to mitigate data analysis variability, MIC values were converted to pMIC, defined as the negative base-10 logarithm of the MIC value.

### 4.2. Molecular Descriptor Calculation and Feature Selection

Padelpy [39] was employed to generate two types of molecular fingerprints for the 353 inhibitors: Molecular ACCess System (MACCS; 166-bit) and PubChem (881-bit). Given the dataset size (~300 compounds), these lower-dimensional fingerprints were chosen over higher-dimensional alternatives like Extended-Connectivity Fingerprints (ECFP; 1024-bit) to prevent overfitting. Feature selection using a variance threshold (<0.1) and Pearson correlation (>0.8) analysis reduced the MACCS and PubChem fingerprint sets to 46 and 41 molecular features, respectively.

### 4.3. ML-QSAR Model Development and Evaluation

Both fingerprint datasets were partitioned into training and test sets using an 8:2 split ratio, resulting in 282 molecules for training and 71 for testing. SVM, ANN, and RF algorithms were implemented to develop individual QSAR models [40] for each fingerprint dataset using the scikit-learn Python(3.9) library.

Following initial model development, PCA was performed to evaluate compound distribution and identify potential outliers in both the training and test sets. PCA was used to transform the high-dimensional descriptor space into principal components, conserving maximum variance in a reduced-dimensionality space [41]. The two leading principal components were then visualized via a scatter plot to identify points deviating from the principal distribution. Any molecules located outside the main data clusters were categorized as outliers and eliminated from the modeling process.

After outlier removal, the models were retrained using grid-based hyperparameter tuning and cross-validation. To determine the best-performing models, a comprehensive suite of statistical metrics was calculated for each model, including RMSE, MSE, MAE, Rp, and 10-fold cross-validation metrics.

### 4.4. Feature Elucidation of the ML-QSAR Model

To enhance the interpretability of the ML-QSAR models, diverse feature importance analysis methods were utilized, including VIP, correlation matrix analysis, and SHAP. For VIP plot generation, the Partial Least Squares Regression (PLSR) method was employed to rank descriptors based on their model contribution [42]. A pairwise correlation matrix was generated to identify molecular fingerprints that were positively or negatively correlated with activity for each model. For the SHAP analysis, SHAP values were computed for each feature to clarify its influence on the pMIC values across the datasets [43].

In addition, a Tanimoto Coefficient-based similarity analysis was conducted to cluster both highly active (pMIC ≥ 7) and weakly active (pMIC ≤ 4) molecules. This clustering approach aided in identifying common molecular features among compounds displaying strong or weak inhibitory activity. To further enhance interpretability, the WordCloud technique was employed to visualize the most common molecular features within these two groups.

### 4.5. ADME-Drug-like Property Analysis

To evaluate the drug-likeness of candidate molecules, a comprehensive pharmacokinetic evaluation of ADMET was conducted using the QikProp module in the Schrödinger (Version 2022-04) software suite. Analysis results showed all prioritized compounds complied with Lipinski’s rule of five with no more than four violations [44].

### 4.6. Molecular Docking

Molecular docking was performed to investigate residue interactions and predict binding energies of lead compounds. For this study, a high-resolution structure of the drug target from acne-related pathways (PDB ID: 8CVM) containing a co-cryo-EM ligand was acquired from the PDB. Missing residues were restored using Glide after removing existing ligands and adding hydrogen atoms. To define the docking target structure, the co-crystallized ligand was used as the center and all structures within a 40 Å radius were selected for truncation as the receptor model. The docking protocol was performed in three steps: protein energy reduction using the Protein Preparation Wizard tool, optimization, and preprocessing to generate the final protein structures. Ligands were prepared using LigPrep, which ensured proper assignment of atom types and protonation states at pH 7.4 ± 1.0. Finally, a grid was defined at the binding pocket coordinates (x, y, z), aligned with the co-crystallized ligand using the Receptor Grid Generation tool.

### 4.7. Experimental Antimicrobial Susceptibility

*C. acnes* strains (American Type Culture Collection; ATCC 6919 and 11827) were obtained from the Guangdong Microbial Culture Collection (GDMCC, Guangzhou, China). For culture preparation, the *C. acnes* strains were cultured on Blood agar in an anaerobic environment at 37 °C for 72 h using a Gaspak system.

The MIC was determined according to the 2018 Clinical and Laboratory Standards Institute (CLSI) guidelines [45]. In a 96-well microtiter plate, 100 µL of various concentrations of test materials (twofold dilutions from 64 to 0.125 µg/mL) were prepared in Müller-Hinton broth containing 1% Dimethyl Sulfoxide (DMSO). Subsequently, 100 µL of bacterial suspension was added to each well to achieve a final volume of 200 µL and a cell density of 10^5^ Colony Forming Units (CFU)/mL. The plates were incubated under anaerobic conditions at 37 °C for 72 h. The MIC was recorded as the lowest concentration of test material permitting no visible growth when compared with control tubes. All experiments were performed in triplicate.

## 5. Conclusions

Our study demonstrates that coupling ML-QSAR with structure-based virtual screening efficiently identifies anti–*C. acnes* agents targeting the 50S ribosomal subunit. Using two complementary molecular representations—MACCS fingerprints modeled by an ANN and PubChem fingerprints modeled by an RF—we achieved strong training performance and consistent external generalization (test Rp > 0.75 for both models). Sequential decision steps—consensus ML enrichment, ADMET triage, docking and interaction analysis, and diversity-preserving clustering—yielded a small, chemically diverse set for testing and produced six experimental hits from a 186,659-compound library. By sampling 60 distinct chemotype clusters, this phased approach ensures comprehensive coverage while enabling focused follow-up on active clusters.

The identification of tripterin represents significant progress in addressing the antimicrobial resistance crisis in acne treatment. Its natural origin, favorable pharmacokinetic properties, and potential to circumvent existing resistance mechanisms are particularly notable. This computational framework demonstrates an efficient approach for antimicrobial discovery from large chemical libraries. Tripterin exhibits a promising dual-action profile, combining direct antimicrobial efficacy through putative 50S ribosomal inhibition with targeted anti-inflammatory activity via NF-κB pathway suppression. This unique combination positions tripterin as a strong candidate for a next-generation therapeutic that can comprehensively address the multifactorial nature of acne.

## Figures and Tables

**Table 1:** Computational profiles of six candidates selected by the screening pipeline, including ML-QSAR pMIC predictions, ADMET descriptors, docking scores against 50S (PDB 8CVM), and cluster IDs.

Methods		Compounds					
		EC23	Carbenoxolone Disodium	Tripterin	3-Oxo-5β-Cholanoic Acid	Glucotropaeolin Potassium	Kaurenoic Acid
ML-QSAR	pMIC MACCS	7.15	7.3	7.32	7.9	8.03	7.36
	pMIC Pubchem	6.2	6.11	6.49	6.93	6.37	6.83
ADMET prediction	MW	332.4	570.8	450.6	374.6	409.4	302.5
	logPo/w	5.82	5.54	5.03	4.68	−0.72	4.69
	PSA	49.4	145.5	89	78.2	181.2	39
	Accept HB	2	8	5	4	14	2
	Donor HB	1	2	2	1	5	1
	logHERG	−4.03	−0.53	−2.35	−1.94	−3.82	−1.3
	#metab	1	4	2	3	6	2
	CNS	−1	−2	−2	−2	−2	−1
	%Human oral absorption	91.1	42.6	76.8	89	28.1	100
	Violation of RO5	1	2	1	0	0	0
Docking	Docking score	−10.9	−10.6	−10.5	−10	−9.4	−9.3
Clustering	Cluster No	19	28	25	39	51	33

**Table 2:** MIC values (µg/mL) of the six screened candidates against *C. acnes* strains ATCC 6919 and ATCC 11827, with clindamycin as a reference control.

Compound Name	MIC (μg/mL)	
	*C. acnes ATCC 6919*	*C. acnes ATCC* 11827
EC23	8	8
Carbenoxolone disodium	>64	>64
Tripterin	2	0.5
3-Oxo-5β-cholanoic acid	>64	>64
Glucotropaeolin Potassium	>64	>64
Kaurenoic acid	8	8
Clindamycin	0.25	0.125

## Data Availability

The data used for model training was obtained from the ChEMBL database https://www.ebi.ac.uk/chembl (accessed on 14 July 2025). The virtual screening library was curated from the ZINC database (https://zinc.docking.org/). The 3D structure of the *C. acnes* 50S ribosomal subunit was retrieved from the Protein Data Bank (PDB ID: 8CVM). All other data generated and analyzed during this study are included in this published article or its supplementary information files. Open code available in https://github.com/jixing475/qsar50s (accessed on 12 November 2025).

## Supplementary Materials

The following supporting information can be downloaded at: https://www.mdpi.com/article/10.3390/molecules30224433/s1, Table S1: Statistical metrics of all the generated MACCS fingerprint-driven QSAR models; Table S2: Statistical metrics of all the generated Pubchem fingerprint-driven QSAR models; Table S3: Chemical definitions and structural interpretations of molecular fingerprint features identified in feature importance analysis; Table S4: Comprehensive molecular interaction profiles of six screened compounds with bond distances and interaction types; Figure S1: Redocking validation of the experimental complex; Figure S2: Binding mode interactions of three inactive compounds (carbenoxolone disodium, 3-oxo-5β-cholanoic acid, and glucotropaeolin potassium).

## Abbreviations

The following abbreviations are used in this manuscript:

ADMET	Absorption, Distribution, Metabolism, Excretion, and Toxicity
ANN	Artificial Neural Network
ATCC	American Type Culture Collection
CFU	Colony Forming Unit
CLSI	Clinical and Laboratory Standards Institute
CNS	Central Nervous System
DMSO	Dimethyl Sulfoxide
GDMCC	Guangdong Microbial Culture Collection
IL-6	Interleukin-6
logHERG	Logarithm of the half maximal inhibitory concentration for the hERG channel
logP	Logarithm of the Octanol-Water Partition Coefficient
MAE	Mean Absolute Error
MACCS	Molecular ACCess System
MIC	Minimum Inhibitory Concentration
ML	Machine Learning
MSE	Mean Squared Error
NF-κB	Nuclear Factor kappa-light-chain-enhancer of activated B cells
PCA	Principal Component Analysis
PDB	Protein Data Bank
PLSR	Partial Least Squares Regression
pMIC	Negative base-10 logarithm of the Minimum Inhibitory Concentration
PSA	Polar Surface Area
QSAR	Quantitative Structure–Activity Relationship
RBF	Radial Basis Function
ReLU	Rectified Linear Unit
RF	Random Forest
RMSE	Root Mean Squared Error
SHAP	Shapley Additive Explanations
SMILES	Simplified Molecular Input Line Entry System
SVM	Support Vector Machine
TNF-α	Tumor Necrosis Factor-alpha
VIP	Variance Importance in Projection
