# Supplementary material for: Integrated Machine Learning and Structure-Based Virtual Screening Identifies Natural Product Targeting 50S Ribosome Inhibitory Activity Against Cutibacterium acnes
Source: Molecules. 2025 Nov 16;30(22):4433. doi: 10.3390/molecules30224433 (PMC12655229; doi:10.3390/molecules30224433)
Supplement: Supplementary file 1 [file molecules-30-04433-s001.zip › molecules-3946905-supplementary.pdf]

Table S1. Statistical metrics of all the generated MACCS fingerprint-driven QSAR models. Best model is in bold.

Model_Type	Algorithm	Train RMSE	Test RMSE	CV RMSE	Train MSE	Test MSE	Train MAE	Test MAE	Train R^2	Test R^2	CV R^2	Train Pearson	Test Pearson
Model (default parameters)	ANN	0.4303	1.1083	0.9968 ± 0.0524	0.1852	1.2284	0.2456	0.8484	0.9314	0.4776	0.6065 ± 0.0450	0.9652	0.7298
	Random Forest	0.5165	1.0245	0.9530 ± 0.0364	0.2668	1.0496	0.3626	0.7862	0.9012	0.5536	0.6447 ± 0.0312	0.9525	0.7579
	SVM	0.8682	1.1682	1.1189 ± 0.0391	0.7538	1.3647	0.5595	0.8898	0.7208	0.4196	0.5164 ± 0.0377	0.8577	0.6797
Model (outliers removed and hypertuned)	**ANN**	**0.4395**	**1.0409**	**1.0601 ± 0.0742**	**0.1932**	**1.0836**	**0.2647**	**0.7980**	**0.9283**	**0.5535**	**0.5498 ± 0.0631**	**0.9637**	**0.7665**
	Random Forest	0.5196	1.0542	0.9740 ± 0.0511	0.2700	1.1113	0.3664	0.8151	0.8998	0.5420	0.6246 ± 0.0385	0.9524	0.7575
	SVM	0.8628	1.1664	1.1179 ± 0.0593	0.7444	1.3605	0.5582	0.8907	0.7237	0.4394	0.5163 ± 0.0403	0.8586	0.6909

Table S2. Statistical metrics of all the generated Pubchem fingerprint-driven QSAR models. Best model is bold.

Model_Type	Algorithm	Train RMSE	Test RMSE	CV RMSE	Train MSE	Test MSE	Train MAE	Test MAE	Train R^2	Test R^2	CV R^2	Train Pearson	Test Pearson
Model (default parameters)	ANN	0.4461	1.1868	1.0750 ± 0.0520	0.1990	1.4086	0.2654	0.8685	0.9263	0.4010	0.5436 ± 0.0486	0.9626	0.6851
	Random Forest	0.5267	1.0705	0.9658 ± 0.0389	0.2774	1.1460	0.3649	0.7557	0.8973	0.5126	0.6358 ± 0.0367	0.9491	0.7391
	SVM	0.8613	1.0511	1.0722 ± 0.0252	0.7418	1.1047	0.5451	0.7872	0.7252	0.5302	0.5566 ± 0.0247	0.8594	0.7448
Model (outliers removed and hypertuned)	ANN	0.4884	1.0734	1.0644 ± 0.0659	0.2386	1.1521	0.3160	0.8027	0.9121	0.4991	0.4932 ± 0.0794	0.9569	0.7276
	**Random Forest**	**0.5257**	**1.0149**	**0.9850 ± 0.0677**	**0.2763**	**1.0301**	**0.3632**	**0.7524**	**0.8982**	**0.5521**	**0.5589 ± 0.0769**	**0.9509**	**0.7564**
	SVM	0.8711	1.0443	1.1032 ± 0.0495	0.7588	1.0905	0.5243	0.7678	0.7204	0.5259	0.4822 ± 0.0678	0.8552	0.7393

Table S3. Chemical definitions and structural interpretations of molecular fingerprint features identified in Figures 3 and 4. Features are organized by fingerprint type (MACCS keys followed by PubChem fingerprints) with their corresponding chemical meanings, SMARTS patterns where applicable, and activity correlations derived from Pearson correlation and SHAP analyses.

Type	Fingerprint ID	Chemical Definition	SMARTS/Pattern	Activity Correlation
**MACCS**	MACCSFP74	CH3 adjacent to CH3	CH3ACH3	Neutral
MACCS	MACCSFP75	Aromatic/aliphatic chain boundary with nitrogen	A!N$A	Neutral
MACCS	MACCSFP80	Nitrogen chain with 3+ atoms	NAAAN	Negative
MACCS	MACCSFP90	Heteroatom-CH2 linkage pattern	QHAACH2A	Negative
MACCS	MACCSFP91	Extended heteroatom-CH2 linkage	QHAAACH2A	Negative
MACCS	MACCSFP95	Nitrogen-oxygen chain pattern	NAAO	Negative
MACCS	MACCSFP97	Extended nitrogen-oxygen chain	NAAAO	Positive
MACCS	MACCSFP100	Aliphatic CH2 adjacent to nitrogen	ACH2N	Positive
MACCS	MACCSFP104	Heteroatom-CH2 linkage	QHACH2A	Negative
MACCS	MACCSFP108	Extended CH3-CH2 pattern	CH3AAACH2A	Neutral
MACCS	MACCSFP113	Non-aromatic oxygen pattern	Onot%A%A	Negative
MACCS	MACCSFP114	Ethyl substituent	CH3CH2A	Negative
MACCS	MACCSFP116	CH3-CH2 chain pattern	CH3AACH2A	Positive
MACCS	MACCSFP120	Multiple heterocyclic atoms (>1)	HETEROCYCLIC ATOM > 1 (&...)	Neutral
MACCS	MACCSFP123	Acetal/ketal or oxygen-carbon-oxygen linkage	OCO	Positive
MACCS	MACCSFP128	Extended CH2 chain pattern	ACH2AAACH2A	Positive
MACCS	MACCSFP131	Multiple heteroatoms with hydrogen (>1)	QH > 1	Positive
MACCS	MACCSFP132	Oxygen-CH2 linkage	OACH2A	Neutral
MACCS	MACCSFP140	High oxygen count (>3)	O > 3 (&...)	Neutral
MACCS	MACCSFP153	Heteroatom-CH2 pattern	QCH2A	Positive
MACCS	MACCSFP155	Chain CH2 boundary	A!CH2!A	Positive
MACCS	MACCSFP156	Nitrogen with multiple substituents	NA(A)A	Negative
MACCS	MACCSFP162	Aromatic ring system	AROMATIC	Positive
**PubChem**	PubchemFP12	16 or more carbons	>= 16 C	Positive
PubChem	PubchemFP14	1 or more nitrogens	>= 1 N	Neutral
PubChem	PubchemFP15	2 or more nitrogens	>= 2 N	Neutral

PubChem	PubchemFP19	2 or more oxygens	>= 2 O	Neutral
PubChem	PubchemFP20	4 or more oxygens	>= 4 O	Positive
PubChem	PubchemFP143	Any 5-membered ring	>= 1 any ring size 5	Neutral
PubChem	PubchemFP179	Saturated/aromatic 6-membered carbon ring	>= 1 saturated or aromatic carbon-only ring size 6	Negative
PubChem	PubchemFP181	Saturated/aromatic 6-membered heteroatom ring	>= 1 saturated or aromatic heteroatom-containing ring size 6	Neutral
PubChem	PubchemFP192	Three or more 6-membered rings	>= 3 any ring size 6	Positive
PubChem	PubchemFP255	Aromatic ring	>= 1 aromatic ring	Negative
PubChem	PubchemFP308	Hydroxyl group (O-H bond)	O-H	Neutral
PubChem	PubchemFP335	Tertiary carbon with hydrogen	C(C)(C)(C)(H)	Neutral
PubChem	PubchemFP338	Carbon with C, H, and N neighbors	C(C)(C)(H)(N)	Positive
PubChem	PubchemFP346	Carbon with C, H, and O neighbors	C(C)(H)(~O)	Negative
PubChem	PubchemFP376	Carbon with N and aromatic C neighbors	C(~N)(:C)	Neutral
PubChem	PubchemFP380	Carbon with two oxygen neighbors	C(O)(O)	Positive
PubChem	PubchemFP476	Enol ether pattern	C-O-C=C	Neutral
PubChem	PubchemFP493	Aromatic ketone/acyl group	O=C-C:C	Positive
PubChem	PubchemFP516	Simple alkene with hydrogens	[#1]-C=C-[#1]	Neutral
PubChem	PubchemFP535	Simple ketone/carbonyl	O=C-C-C	Neutral
PubChem	PubchemFP566	Amino alcohol pattern	O-C-C-N	Positive
PubChem	PubchemFP580	Amino ketone pattern	O=C-C-C-N	Positive
PubChem	PubchemFP581	Keto-ester/hydroxy ketone pattern	O=C-C-C-O	Neutral
PubChem	PubchemFP696	Long carbon chain (8 carbons)	C-C-C-C-C-C-C-C	Neutral
PubChem	PubchemFP697	Branched long carbon chain	C-C-C-C-C-C(C)-C	Neutral

Note: Activity correlation indicates whether the feature is positively correlated (beneficial for antimicrobial activity), negatively correlated (detrimental to activity), or neutral based on Pearson correlation coefficients and SHAP values from Figures 3 and 4. MACCS keys use a symbolic notation where: A = any atom, Q = heteroatom (non-C, non-H), X = halogen, $ = ring bond, ! = chain bond, % = aromatic bond. PubChem fingerprints use standard SMARTS notation where: ~ denotes any bond, : denotes aromatic bond, - denotes single bond, = denotes double bond, and [#1] denotes hydrogen.

Table S4. Comprehensive molecular interaction profiles of six screened compounds with the *C. acnes* 50S ribosomal subunit PTC pocket

Compound	MIC (μg/mL)	Interaction Type	Residue	Residue Type	DIST_H-A (Å)	DIST_D-A (Å)	Donor Angle (°)	Protein is Donor	Donor Type	Acceptor Type
**Tripterin**	0.5–2	Hydrogen Bond	U2766	U	2.86	3.53	124.17	Yes	Nar	O2
		Hydrogen Bond	U2767	U	2.55	3.44	155.77	No	O2	O3
		Hydrogen Bond	U2767	U	2.18	3.12	165.74	Yes	O3	O2
		Hydrophobic	C2768	C	—	3.46	—	—	—	—
**EC23**	8	Hydrogen Bond	U2766	U	2.05	2.98	152.30	Yes	Nar	O.co2
		Hydrophobic	C2768	C	—	3.83	—	—	—	—
**Kaurenoic acid**	8	Hydrogen Bond	G2687	G	5.28	6.21*	166.98	Yes	O3	O.co2
		Hydrophobic	U2688	U	—	4.58	—	—	—	—
		Hydrophobic	U2767	U	—	6.39	—	—	—	—
**Carbenoxolone disodium**	>64	Hydrogen Bond	G2687	G	1.92	2.91	165.30	Yes	Npl	O.co2
		Hydrogen Bond	U2766	U	2.80	3.68	146.42	Yes	Nar	O2
**3-Oxo-5β-cholanoic acid**	>64	Hydrogen Bond	A2621	A	1.98	2.95	160.22	Yes	Npl	O2
		Hydrogen Bond	G2687	G	2.24	2.94	125.00	Yes	Npl	O.co2
		Hydrogen Bond	U2766	U	1.98	2.95	162.71	Yes	Nar	O.co2
**Glucotropaeolin Potassium**	>64	Hydrogen Bond	C2624	C	1.88	2.71	142.78	No	O3	O2
		Hydrogen Bond	C2768	C	2.24	3.16	163.40	No	O3	O2

Note: Interaction data extracted from PLIP (Protein-Ligand Interaction Profiler) analysis of docked poses. For hydrogen bonds: DIST_H-A represents hydrogen-to-acceptor distance, DIST_D-A represents donor-acceptor distance, and donor angles indicate hydrogen bond geometry. For hydrophobic interactions: DIST_D-A represents the contact distance between carbon atoms; other hydrogen bond-specific parameters are not applicable (—). *Donor-acceptor distance of 6.21 Å indicates a weak hydrogen bond interaction.

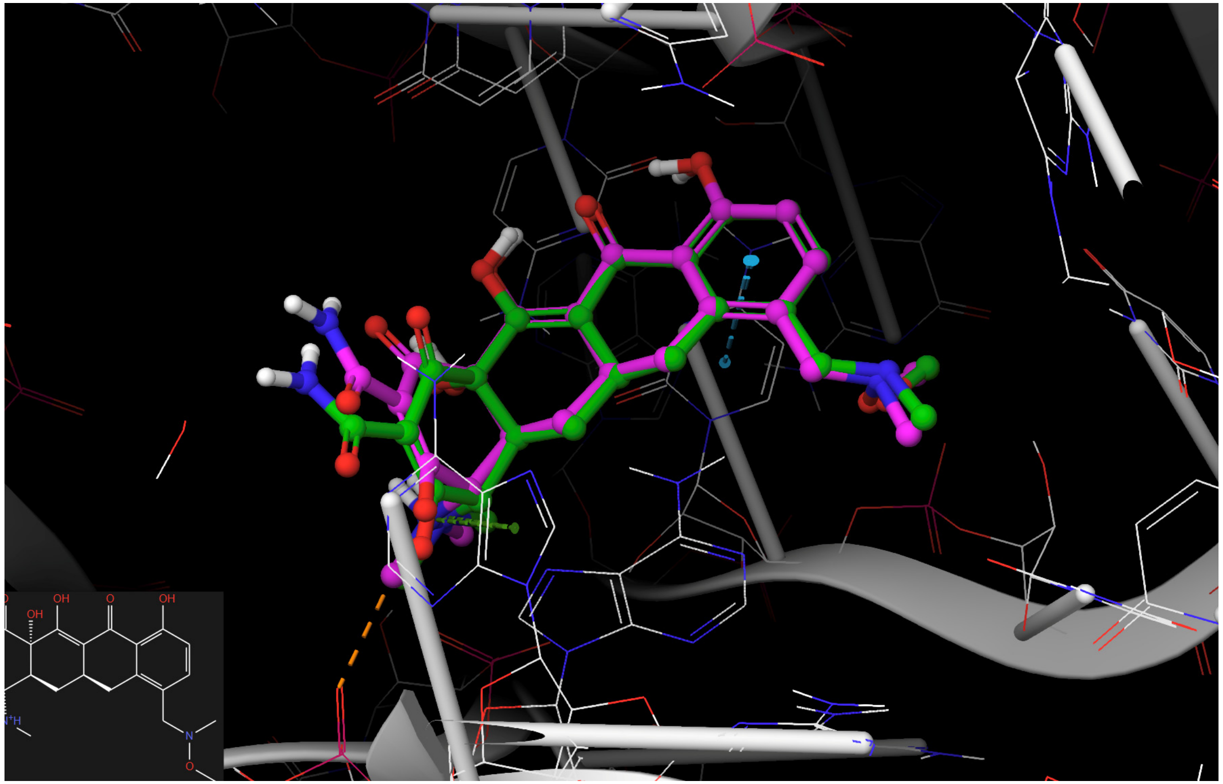

Figure S1: Redocking validation of the co-crystallized complex, demonstrating an RMSD of 0.66 Å (Green is the crystallographic reference conformation).

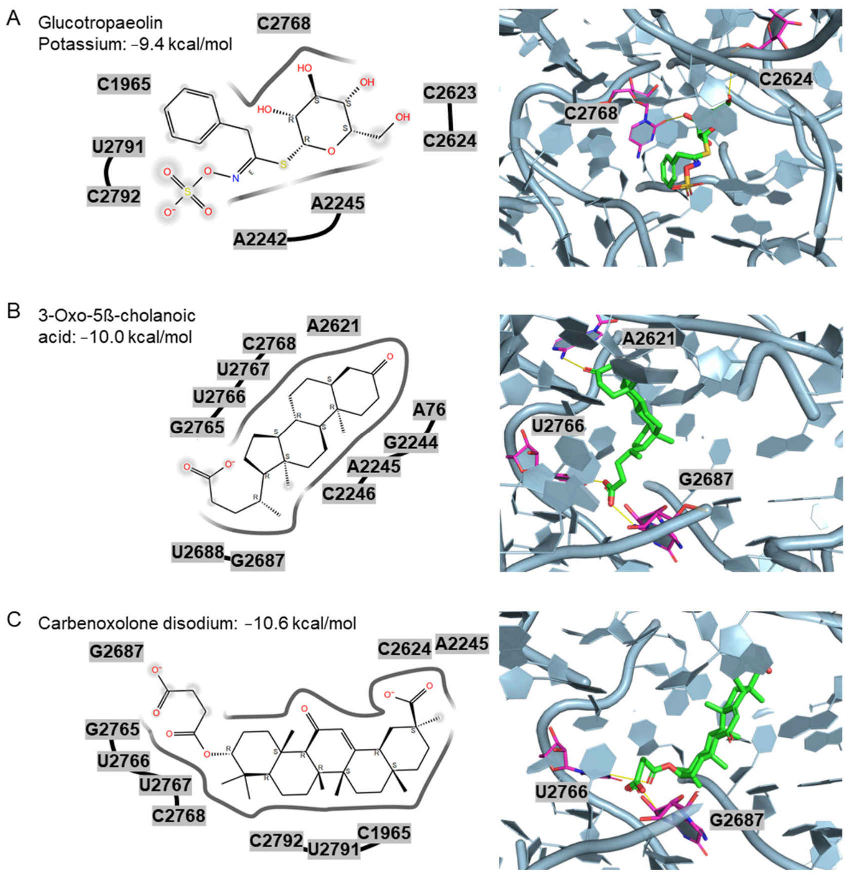

Figure S2: 2D and 3D docking interactions of inactive compounds within the PTC pocket of the 50S ribosomal subunit.
